# Cold Brew Coffee: Consumer Acceptability and Characterization Using the Check-All-That-Apply (CATA) Method

**DOI:** 10.3390/foods8080344

**Published:** 2019-08-13

**Authors:** JeongAe Heo, Kap Seong Choi, Shangci Wang, Koushik Adhikari, Jeehyun Lee

**Affiliations:** 1 Department of Food Science and Nutrition & Kimchi Research Institute, Pusan National University, Busan 46241, Korea; 2 Korea Food Research Institute, Wanju 55365, Korea; 3 School of Food Science, Sunchon National University, Suncheon 57922, Korea; 4 Department of Food Science and Technology, University of Georgia, Griffin, GA 30223, USA

**Keywords:** coffee, cold brew, CATA, consumer acceptability

## Abstract

The aim of this study was to investigate consumers’ acceptability and perceived sensory attributes of cold brew coffee, which is increasing in popularity. A total of 120 consumers evaluated liking of 13 cold brew coffee samples and checked sensory attributes they perceived using the check-all-that-apply (CATA) method. Correspondence analysis identified characteristics of each cold brew sample and brewing methods, namely cold brew, coffee machine brewed but served cold, ready-to-drink, and purchased from a coffee shop. In addition, a reduced number of terms were reviewed for common-to-all cold brew samples (17 terms) and specific to each sample (48 terms), which also discriminated among samples. Furthermore, data on consumers’ liking were not influenced by caffeine contents and most of the volatile compounds, but chlorogenic acid and trigonelline contents were negatively related with sensory data. This study specifies the characteristics of cold brew coffee using the CATA method, shows consumers’ segmentation using acceptability, and investigates the relationship between sensory liking data and non-volatile, volatile compounds of coffee.

## 1. Introduction

Coffee is one of the most popular beverages, which has various types and flavor. In Korea, coffee consumption per capita is increasing steadily every year and consumers’ needs are diversifying. Similarly, the Korean market for cold brew coffee is also expanding, and the various types of ready-to-drink (RTD) and cold brew coffees are now available in coffee shops. Due to its complex flavor and characteristics, various studies on coffee have been conducted on instrumental analysis [1], functional properties [2], and sensory aspects [3,4]. The sensory attributes of coffee are influenced by many factors such as coffee species and varieties, roasting methods and temperature, extraction methods, packaging, and preparation [5,6]. However, most sensory research studies for coffee focuses on hot brewed or espresso by a coffee maker. Furthermore, much research has also been conducted to identify volatile coffee compounds [7,8] and non-volatile compounds that contribute to aroma perception, astringency and bitter taste, respectively [9,10].

According to study of Angeloni et al. [11], sensory characteristics of coffee by extraction methods (drip, cold brewing, and French press) were different, and these different sensory characteristics were thought to be the result of the extraction process or temperature. However, few studies have considered cold brew coffee in terms of both instrumental and sensory aspects. Therefore, it is important to examine and identify consumers’ acceptability of cold brew coffee and the sensory attributes that affect perception and interaction between them. Moreover, various types of cold brew coffee (e.g., cold brew coffee from coffee shop, ready-to-drink products, and home-brewed) that consumers can experience should be considered to provide fundamental information about cold brew coffees.

The check-all-that-apply (CATA) method is widely used to investigate sensory perception. It is a relatively low cost and time saving method compared to descriptive analysis and can obtain rapid responses from consumers [12,13]. Because this methodology can collect intuitive responses from consumers, many consumer tests have used the CATA to evaluate fruit-flavored powder [14], ice cream [15], milk desserts [16], and orange juice [17].

The objectives of this study were to (1) understand consumers’ acceptability of various types of cold brew coffee, (2) determine the sensory characteristics of each cold brew coffee sample using the CATA method, (3) explore the different sensory characteristics of general hot water extraction and cold brew coffee samples to examine the influence of extraction temperature, and (4) investigate the correlation between consumer perception and the non-volatile and volatile compounds of coffee samples.

## 2. Materials and Methods

### 2.1. Sample Preparation and Presentation

Thirteen coffee samples were used in this study, including one blind duplicate (Table 1). To minimize fatigue and non-motivation due to the number of sensory attributes for the CATA, the experiments were split into two sessions, and seven samples were provided in each session. All samples were stored in a refrigerator at 4 °C to maintain the temperature and served at about 8 ± 2 °C. To limit consumption of too much caffeine (less than 400 mg per day for adults according to the Ministry of Food and Drug Safety [18]), each sample (50 mL) was provided in a clear plastic cup (198 mL, TNS sunflower, Incheon, Korea) coded with a three-digit random number. Samples were served every seven minutes and presented according to a William’s Latin Square design. Unsalted crackers and bottled water were also provided to cleanse the palate between samples.

#### 2.1.1. Brewed Coffee

Three types of coffee beans and Folger classic roast (Folgers, Orrville, OH, USA) were used for the brewed coffee (Table 1). Coffee beans were purchased from local coffee shops and ground with a medium setting (Delonghi KG79, Treviso, Italy). Both the cold brew and general brew (made in a coffee maker) coffees were made with 7 g of ground coffee in 100 mL of water. For the cold brew coffee, ground coffee was first placed in a glass bottle (standard wide-mouth bottles with polyvinyl-lined caps, Fisher Scientific, MA, USA), then water was poured over it, after which it was brewed for nine hours under refrigerated conditions at 4 °C. Coffee was first filtered through a stainless steel filter (Kinto, Hikone-shi, Shiga, Japan) and then re-filtered through a paper filter (Mellitta paper filter 1 × 4, Mellitta, Minden, North Rhine-Westphalia, Germany) to remove fine particles. The filtered brewed coffee was stored in a refrigerator (4 °C) until used, which was within 22 h of brewing. The general brewed coffee was made in a coffee maker (Phillips HD-7564, Amsterdam, The Netherlands), and filtered through a paper filter (Mellitta paper filter 1 × 4). Brewed coffee was made at least two hours before the evaluation and refrigerated until served after cooling down in an ice bath for 30 min. These samples were used within 10 h of brewing.

#### 2.1.2. Ready-to-Drink Coffee

Ready-to-drink coffee samples—French Café cold brew coffee (Namyang, Seoul, Korea) and Barista Rules cold brew coffee (Maeil, Seoul, Korea)—were purchased from a convenience store (GS retail, Busan, Korea). The cold brew by Babinski coffee (Korea Yakult, Seoul, Korea) was delivered on the morning of every evaluation day. Samples were kept refrigerated and taken out just before serving.

#### 2.1.3. Coffee from a Coffee Shop

Cold brew coffees were purchased from two local coffee shops located in Busan, namely Starbucks (Seattle, WA, USA) and Twosome Place (CJ Foodville, Seoul, Korea). Samples were purchased on the morning of evaluation, without ice to prevent gradual dilution, kept refrigerated, and used within eight hours.

### 2.2. Consumers’ Evaluation Procedure

This study was approved (IRB 2016_49_HR) by the institutional review board of Pusan National University, and subject’s consent for participation was obtained. In total, 120 naïve coffee consumers who were not pregnant, allergic to food, or sensitive to caffeine participated and evaluations were conducted in individual sensory booths. The questionnaire was composed of items pertaining to acceptability, perceived intensity, and perceivable sensory attributes of the samples. Therefore, overall, sourness, bitterness, and coffee flavor (coffeeID) acceptability were evaluated using nine-point hedonic scale and perceived sourness, bitterness, and coffeeID intensity were measured using nine-point category scale.

To identify consumers’ acceptability and perceived intensity of cold brew coffee products, the CATA method was applied using 108 sensory attributes of coffee [19]. Terms were translated into Korean and back translated to English by another researcher to determine proper translation [20]. All terms were translated into plain terms as possible, which consumers could easily understand and Korean words that convey original meaning in English were selected. Basic demographic information was also included in questionnaire. To prevent the possibility of light insomnia due to coffee tasting, evaluation sessions were conducted between 10 a.m. and 4 p.m.

### 2.3. Instrumental Analysis

Volatile compounds affect coffee aroma, while non-volatile compounds such as caffeine, trigonelline, and chlorogenic acid are important contributors to coffee’s bitterness or astringency. The relationship between consumers’ perception and non-volatile and volatile compounds based on data collected using high performance liquid chromatography (HPLC) and headspace-solid phase micro extraction (HS-SPME), gas chromatography–mass spectrometry (GC/MS) were determined (detailed data were not listed).

#### 2.3.1. Non-Volatile Compounds (HPLC)

Samples were treated with Carrez reagents I and II and 0.3 mL of them was mixed 10 mL of each samples for caffeine. After being centrifuged (10 min at 45,000× *g*), supernatant was filtered (0.45 µm membrane filter, Acrodisc Syringe Filter, Gelman Sciences, Ann Arbor, MI, USA) and XBridge™ Shield RP18 (5 µm, 4.6 mm × 250 mm, Waters, Milford, MA, USA) was used for analysis. Ten millimolar of citric acid and methanol were used for mobile A and B, respectively and the detector was set at 276 nm. In case of chlorogenic acid, samples were treated in the same way of caffeine except detector was set at 325 nm. For trigonelline, each 10 mL of samples was mixed with 0.3 mL of Carrezz reagents I and II and 0.6 mL of absolute methanol (HPLC-grade, Merck, Damstadt, Frankfurter, Germany). Other processes were identical with caffeine and chlorogenic acid except that a reverse-phase column (XBridge™ Shield RP18; 5 µm, 4.6 mm × 150 mm, Waters, Milford, MA, USA) and 0.5% methanol (HPLC-grade, Merck, Damstadt, Frankfurter, Germany) was used. The flow rate was 0.8 mL/min and the detector was set at 264 nm.

#### 2.3.2. Volatile Compounds (HS-SPME, GC/MS)

Two milliliters of coffee samples was contained in a 10 mL of vial with silicon/ polytetrafluoroethylene (PTFE) septum (18 mm diameter × 3.2 mm thickness, Varian, Inc., Walnut Creek, CA, USA) and 5 µL of 0.045 mg/mL 1,3-dichlorobezene (Sigma-Aldrich, St. Louis, MO, USA) solution was added to the sample as an internal standard. After equilibration for 10 min at 60 °C at 250 rpm, a 50/30 µm three phase (divinylbenzene/carboxen/polydimethylbenzene, DVB/CAR/PDMS) SPME fiber (Supelco, Bellefont, PA, USA) was used to extract volatile compounds at 60 °C for 45 min. After that, fiber was desorbed into the injection port of a GC/MS (Model 7890A, Agilent Technologies, Santa Clara, CA, USA) equipped with spectrophotometer detector (Model 5977A, Agilent Technologies, Santa Clara, CA, USA). A HP-5 ms column (30 m × 250 µm × 0.25 µm thickness, Agilent Technologies, Santa Clara, CA, USA) was used to separate the analytes. Initial temperature of the column was 40 °C for 10 min and changed to 8 °C/min to 180 °C, 10 °C/min to 280 °C, and held for 10 min at 280 °C. The MS detector scanned a mass range (m/z) from 30 to 400 m/z with scan speed 1.562 µ/s. Identification of compounds was based on both the mass spectra database (NIST/EPA/NIH mass spectral library, Version 2.2, 2014). Semi-quantification was done for the identified compounds, and the relative concentration was reported based on the area of the 1,3-dichlorobezene, which was used as the internal standard. Three replications were carried out.

### 2.4. Statistical Analysis

Liking scores and perceived intensity scores were analyzed using one-way analysis of variance (ANOVA) to determine any significant differences among samples. Fisher’s least significant difference (LSD) was conducted where significant difference was found. In addition, Pearson’s correlation analysis was used to investigate the linear relationship between two variables [21]. Data from the CATA was presented as the frequency of checked sensory attributes, and correspondence analysis (CA) was conducted to visually compare differences among samples and sensory attributes. Ward’s minimum variance cluster analysis was also conducted to segment consumers according to their overall acceptability ratings. The aforementioned analyses were conducted using SAS 9.4 (SAS Institute Inc., Cary, NC, USA). To explore the relationship between variables such as consumers’ acceptability and components of coffee, multi-factor analysis (MFA) were employed using XLSTAT 19 (Addinsoft, New York, NY, USA).

## 3. Results and Discussion

### 3.1. Consumers’ Demographic Information

A total of 120 consumers participated and of these, 58 were male and 103 (86%) were aged 19–25 years old and 111 participants were students. Consumers’ consumption behaviors are shown in Table 2. About 76% of participants responded that they consumed cold black coffee more than once a week. Evaluation was conducted in August (average high 33.4 °C in Busan), so there may be the possibility of seasonal influence on the frequency of cold black coffee consumption. 

Both hot and cold black coffee were consumed at similar frequencies: 52% and 64% of participants consumed hot or cold black coffee in the afternoon, respectively. When selecting coffee, consumers tended to care about taste of coffee the most. A total of 90% cared about its flavor, and 77% about its aroma. Degree of bitterness and price were also important factors. Finally, most consumers (about 87%) responded that their favorite type of coffee was coffee shop coffee.

### 3.2. Overall Liking and Perceived Sensory Intensity Scores

Consumers’ acceptability and perceived sensory intensity mean scores are shown in Table 3. BaristaRTD(A) and its blind duplicate, BaristaRTD(B), scored similarly in all liking and intensity categories, and no significant differences (*p* > 0.05) between them were evident. This indicates that comparing consumers’ evaluations from two different sessions was reliable. However, mean liking scores were generally low, ranging between “dislike moderately” to “like slightly.” BaristaRTD ranked the highest for liking in terms of overall liking, bitterness and sourness liking while the BabinskiRTD sample scored highest for coffeeID liking. Overall, the liking scores for BabinskiRTD and BaristaRTD were relatively higher, whereas the cold brew (CB) and coffee maker (CM) samples scored lower. Specifically, KenyaCM scored the lowest in every liking category, and consumers gave the highest sourness intensity to Colombia and Kenya samples. FolgersCM samples scored the highest for bitterness intensity. The highest perceived sourness intensity of Kenya samples might have decreased consumers’ sourness liking. Regarding coffeeID intensity, the CS sample scored the highest, whereas RTD samples received relatively lower scores.

Consumers were asked to freely express their opinions about the coffee samples as an open-ended comment. The most frequently used words were sourness (386) and bitterness (297), showing that these attributes were dominant characteristics of the coffee samples. Interestingly, oriental medicine was also mentioned about 19 times. The medicinal attribute in the CATA list referred to sterile characteristics such as alcohol and iodine but consumers seemed to consider it as oriental medicine. Korean consumers seemed to taste the herbal medicine attribute when they tried too much bitterness, which could be attributed to the oriental food culture. Korean panel produced and used more particular descriptors about perilla oil than panels from different country [22]. Considering most Koreans have experienced oriental medicine, strong coffee flavor and bitterness may have influenced perception of oriental herbal medicine-like flavor. Since there were no herbal medicine attributes in the CATA questionnaires, there was the possibility of the dumping effect [23] for medicinal attributes or other bitterness-related attributes.

### 3.3. Analysis of the CATA Terms

#### 3.3.1. Correspondence Analysis with 108 Attributes

**Figure 1:**
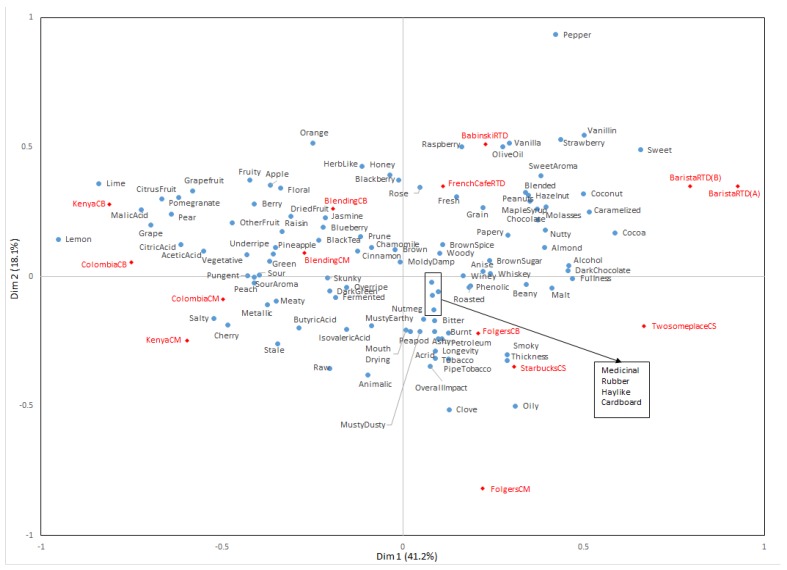
Correspondence analysis of consumers.

Coffee samples and the CATA terms were visually shown through correspondence analysis (Figure 1). Dimensions 1 and 2 explain a total of 59.3%. Dimension 1 was positively associated with caramelized, cocoa, coconut, dark chocolate, and fullness, and negatively related with citrus fruit, grape, lemon, lime, and malic acid attributes. Dimension 2 could be explained positively by blackberry, herblike, orange, raspberry, strawberry, and vanillin attributes, and negatively by animalic, clove, musty/dusty, and tobacco attributes.

RTD samples were positioned in quadrant 1, and Folgers samples and Coffee Shop samples were in quadrant 4. The other Blended, Colombia, and Kenya samples were in quadrants 2 and 3. BabinskiRTD was positioned near olive oil and vanilla attributes and BaristaRTD were associated with coconut and sweet attributes. FrenchCaféRTD was related to fresh, grain, and rose attributes. For Colombia and Kenya beans, brewing methods influenced flavors more than beans, but blending beans may reduce differences obtained from brewing methods. ColombiaCB and KenyaCB were explained mainly by acids and citrus fruit attributes such as acetic acid, citric acid, and malic acid as well as citrus fruit, lemon, and pear. Machine brewed coffees—ColombiaCM and KenyaCM were related to peach and cherry, respectively, and they were also close to pungent, salty, and sour aromas. Blended samples seem to be related to black tea, cinnamon, dried fruit, green, prune, and sour attributes. FolgersCB was situated near acrid, bitter, burnt, pipe tobacco, and tobacco attributes, while FolgersCM was correlated with clove, overall impact, pipe tobacco, smoky, thickness, tobacco, and oily attributes. CS samples were related with overall impact and pipe tobacco, especially, TwosomeplaceCS was related to smoky and thickness attributes. CS samples were typically sold with ice in coffee shops. However, CS samples were provided without ice in this study to prevent dilution. Thus, consumers might have felt the thickness and stronger coffeeID in the CS samples, because it would be more concentrated than the regular iced coffee they consume.

Kim and Kim [24] compared cold brew coffee by dripping methods and cold brew coffee by steeping methods and each sample were extracted 3, 6, 9 and 18 h, respectively. As results of quantitative descriptive analysis (QDA) suggest, acidity, bitterness, and sweetness intensity were different by methods and brewing time. Considering steeping method was used for this study, it seemed different extraction time of each types of samples influenced consumers’ sensory perception.

#### 3.3.2. Frequency Results and Correspondence Analysis of the Reduced Terms

The 108 CATA descriptors [19] were general terms for all coffees, not specific terms for cold brew coffee. Thus, some attributes were not checked or did not contribute towards discriminating among samples. Since the number of attributes for cold brew may be reduced, the frequency of attributes for each sample was investigated to identify unnecessary terms.

Overall, at least 10% of consumers checked 17 attributes (acrid, ashy, bitter, burnt, dark chocolate, grain, hazelnut, longevity, medicinal, mouth drying, musty/earthy, nutty, overall impact, roasted, sour, sour aromatics, and woody) when the sum of frequency of samples by attributes was reviewed. When the frequency of attributes for individual coffee was reviewed, 10% or more of consumers checked 48 attributes (acetic acid, acrid, alcohol, ashy, beany, bitter, blended, brown sugar, burnt, caramelized, chocolate, citric acid, citrus fruit, dark chocolate, fermented, fresh, fruity, fullness, grain, green, hay-like, hazelnut, longevity, malic acid, medicinal, metallic, mouth drying, musty/dusty, musty/earthy, nutty, other fruit, overall impact, over-ripe, petroleum, pipe tobacco, pungent, roasted, salty, sour, sour aroma, stale, sweet, sweet aroma, thickness, tobacco, under-ripe, whisky, and woody) for at least one sample and the frequencies of each attribute were listed by coffee type. CB coffee samples (*n* = 4) had more checks for citric acid, citrus fruit, fruit, and pungent attributes compared to other coffee groups. RTD coffee samples (*n* = 4) were characterized by dark chocolate, fullness, hazelnut, and sweet aromatics and they had fewer checks for mouth drying attributes than other groups. Of all the samples evaluated, the CS coffee group (*n* = 2) was the only group to have caramelized, petroleum, thickness, and whisky checked.

**Figure 2:**
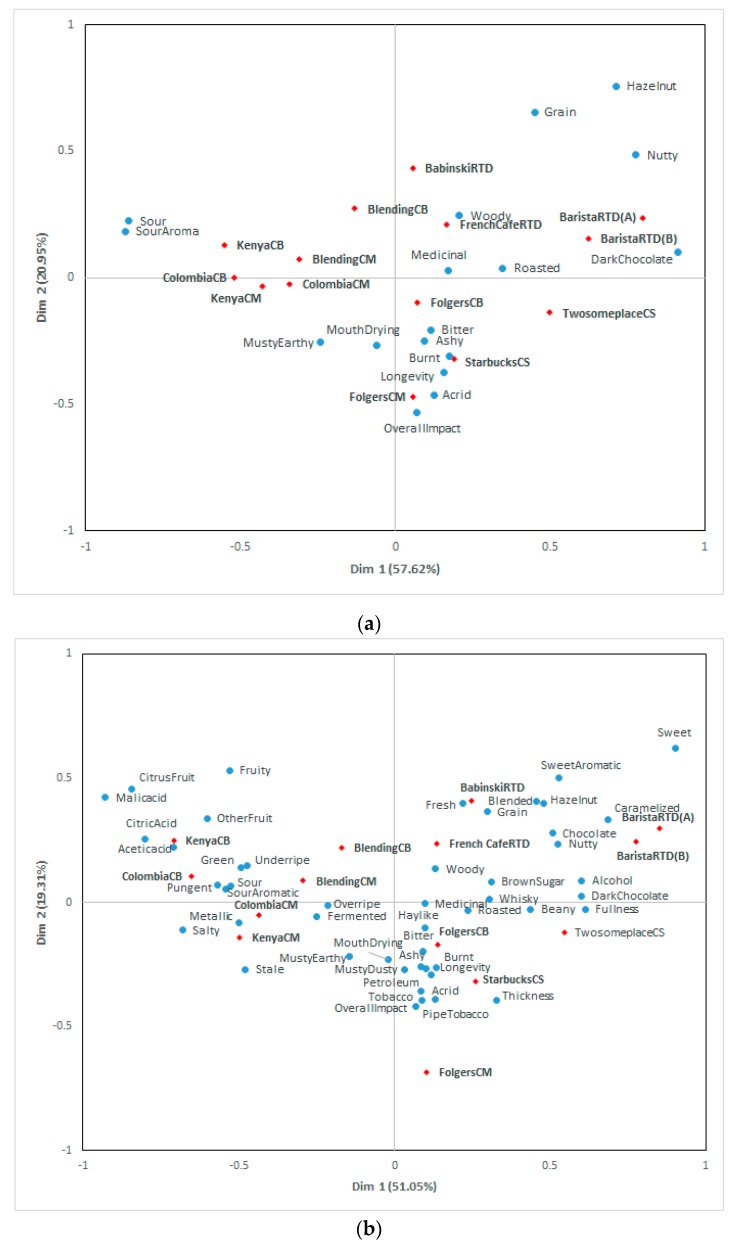
Correspondence analysis bi-plot using 17 commonly selected terms for all cold brew coffee samples by at least 10% of consumers (**a**) and correspondence analysis bi-plot using 48 terms selected for one or more cold brew coffee samples by at least 10% of consumers (**b**).

Additional correspondence analyses were conducted using reduced attributes to investigate if the reduced number of attributes could be used to discriminate among the samples. The results of the correspondence analysis of the CATA terms using 17 and 48 attributes are shown in Figure 2. They explained 78.57% and 70.36%, respectively. All samples were positioned in the same quadrant compared to Figure 1, and the variability explained by the first two dimensions increased. Imamura [25] developed 88-term lexicon for 149 soy sauce products using a trained panel. Nineteen common attributes out of 88 descriptors could be used to differentiate intensities among soy sauce samples, saving time and costs. Similarly, 17 and 48 terms in this study could be utilized in future studies, lowering the time and cost required and still showing discrimination among cold coffee samples.

### 3.4. Cluster Analysis of Consumers

**Figure 3:**
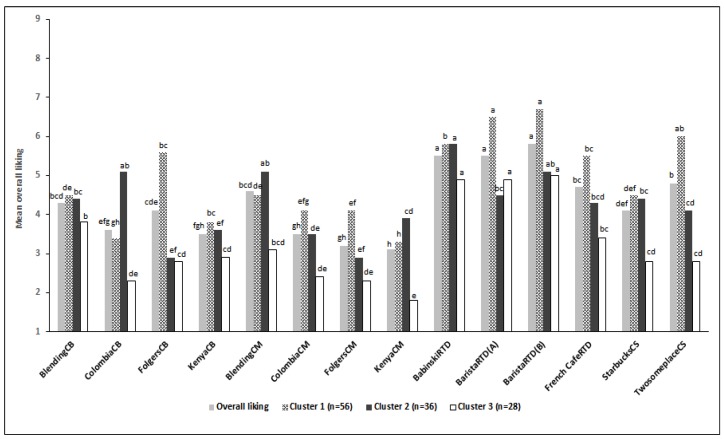
Overall liking of coffee by three consumer clusters.

Based on overall liking scores, three consumer groups were identified (Figure 3). These consumer segments were differed in their overall liking ranges and ranking of their preference. For cluster 1 (*n* = 56), the most-liked samples were BaristaRTD (6.6) followed by TwosomeplaceCS (6.0) and BabinskiRTD (5.8). They preferred TwosomeplaceCS to StarbucksCS, and their least-liked samples were Colombia and Kenya samples. Cluster 2 (*n* = 36) preferred BabinskiRTD (5.7), followed by ColombiaCB and BlendingCM (5.1). They preferred StarbucksCS to TwosomeplaceCS and disliked the Folgers samples. Mean overall liking scores for cluster 3 (*n* = 28) were lower than that of other clusters. The most-liked samples were BaristaRTD (5.0) and BabinskiRTD (4.9) and cluster 3 showed the same liking scores for both StarbucksCS and TwosomeplaceCS. Hughson and Boakes [26] also showed that high consumption group of wine discriminated wine samples based on flavor attributes better than low wine consumption group. In this study, specifically, cluster 2 demonstrated the highest frequency of cold black coffee consumption at 22.2%, followed by cluster 3 (14.3%) and cluster 1 (5.36%). Considering the distinct liking patterns of cluster 2, the frequency of consumption might affect acceptability ranking based on their flavor perception of coffee.

### 3.5. Comparison between Consumer Sensory Perception and Instrumental Analysis—Multi-Factor Analysis (MFA)

**Figure 4:**
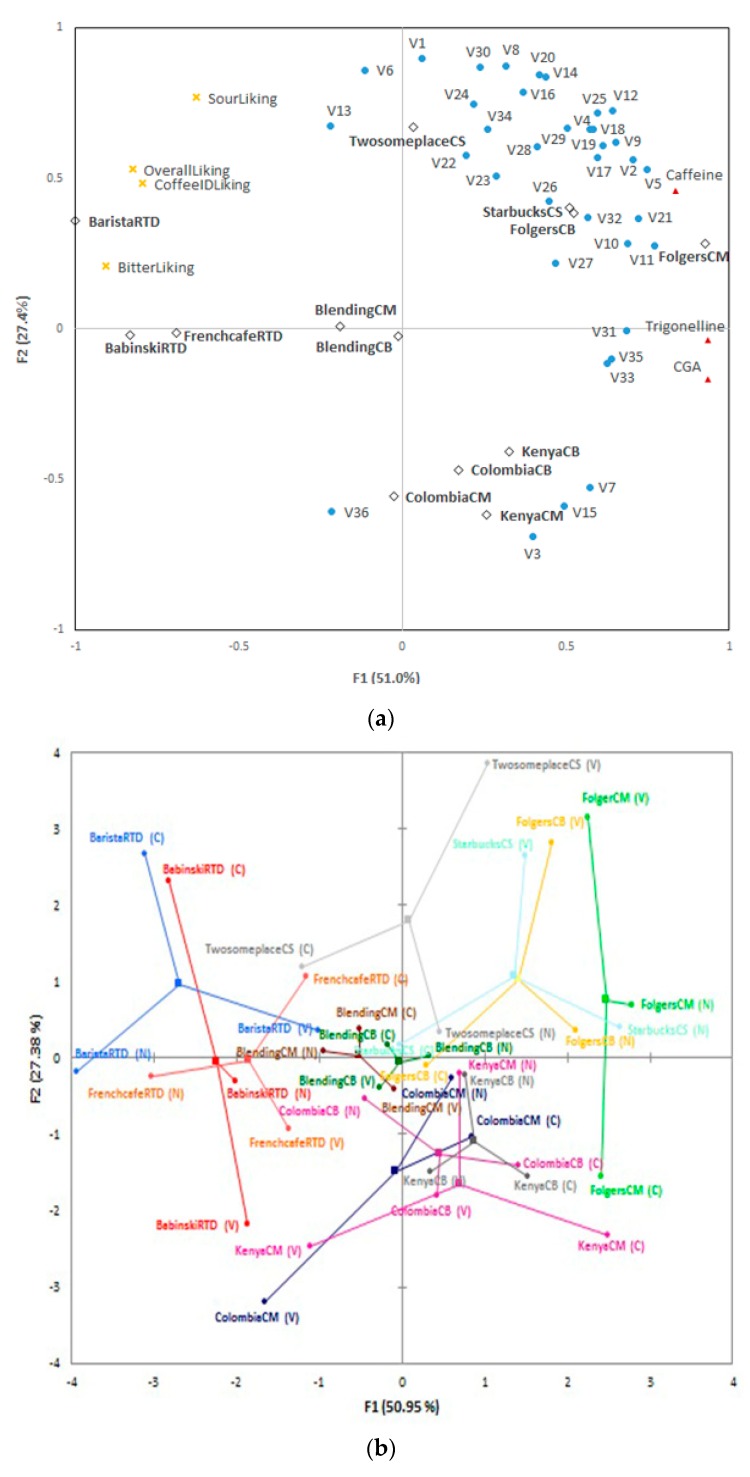
Multi-factor analysis of instrumental and sensory data (**a**) and superimposed map of coffee samples (**b**). (C), (N), and (V) stands for consumer acceptability, non-volatiles compounds, and volatile compounds, respectively.

Multi-factor analysis (MFA) was conducted to illustrate the relationship among multiple variables, such as sensory liking data, instrumental data (36 volatile and 3 non-volatile compounds), and coffee samples (Figure 4a). F1 and F2 explained 78.4% of total variance. The MFA results were similar to those of the correspondence analysis from the CATA, indicating similarity in line with brewing methods, except for blending samples. This may suggest that volatile compounds affect the position of samples on a CA plot (Figure 1) to some degree.

CS and Folgers samples were positioned in quadrant 1 where most volatile compounds were positioned including guaiacol (V20) and 4-ethylguaiacol (V29), which indicates spice and clove, smoke, sweet, and medicine characteristics [27]. On the contrary, other samples such as RTD samples were positioned in quadrants 2 and 3, showing no specific relationship with most volatile compounds. Colombia and Kenya samples were placed roughly in quadrant 4 with furfural (V3), 5-methyl-2-furaldehyde (V7), 3,5-octadien-2-one (V15), and tetradecanoic acid (V36) suggesting they were distinct indicators of these coffee samples.

Caffeine is an alkaloid that has bitter taste [28], and chlorogenic acids has both bitterness and astringent characteristics [9]. Trigonelline is known to influence aroma perception of coffee [29]. Caffeine is close to CS and Folgers samples and to most volatile compounds. Thus, the most volatile compounds and caffeine seemed unrelated with sensory liking data, whereas chlorogenic acid and trigonelline had a slightly inversed relationship with sensory liking data. Contrary to this result, Zanin *et al*. [30] showed that although there was a slightly positive correlation between coffee quality and chlorogenic acid content, but still there were no consistent relationship between coffee cup quality and chlorogenic acid content. They were associated with 4-vinylguaiacol (V31), 3,4-dimethoxystyrene (V33), and dodecanoic acid (V35).

Additionally, blended samples were not explained by specific variables, positioned near the RTD samples. Aroma and flavors of coffee can be balanced by blending and coffee including diverse variety can maintain its quality even if when ingredients undergo changes [31]. Thus, comparing other brewed coffee such as Colombia and Kenya known to have distinct characteristics, blending samples might have relatively balanced characteristics resulted from blending. Besides, although RTD samples were also composed of various variety of coffee beans like the blended samples, thermal process such as sterilization could also influence coffee flavor [32], causing different profiles unlike blending samples.

The superimposed map is presented by colored dots (Figure 4b). Each dot signifies instrumental and sensory data and mean values are also marked among them. The RV coefficient, measuring closeness of two sets of matrix using value between 0 and 1, of consumers and non-volatile compounds, consumers and volatile compounds were about 0.52, 0.27, respectively, and RV coefficient between non-volatile and volatile components was about 0.46. Overall, the arrangement of samples was different by measurement (volatile and non-volatile contents and sensory data) and this result suggested that position of samples was not similar by measurements.

Non-volatile compounds contents were mainly explained by F1. Consumers’ data were explained by both F1 and F2, and consumers’ liking seemed to increase in the direction of quadrant 2. Volatile compounds contents were also explained by F1 and F2, but volatile compounds contents tended to increase toward quadrant 1. This opposite tendency between consumers’ data and volatile compounds and a low RV coefficient (0.27) seemed to be due to the diversity and difference in contents of volatile compounds. The RV efficient could be higher if a few key volatile compounds were used for analysis. However, key volatile compounds of cold brew profiles had not been established due to limited preceding research and should be further researched.

## 4. Conclusions

This study investigated the comprehensive sensory attributes derived from cold brew coffee and consumers’ perception using the CATA method. In addition, three clusters of consumers were identified based on their overall liking scores. The results of a correspondence analysis indicated the relationship between each type of cold brew and specific sensory attributes. Furthermore, a reduced number of terms were commonly reported for all cold brew samples (17 attributes) or at least for one coffee sample (48 attributes) by more than 10% of the consumers. These reduced terms (17 and 48 attributes) also demonstrated reliable discriminating ability among cold brew coffee samples by yielding similar results compared to that of all attributes used. This shortened list of terms and sensory attributes may be more appropriate in evaluating cold brew coffee in the future, especially in further consumer research. In terms of the instrumental aspects, caffeine (*r* = −0.45, *p* = 0.1264) did not have significant correlation with consumers’ overall liking whereas chlorogenic acid (*r* = *−*0.82, <0.0006) and trigonelline (*r* = *−*0.76, *p* < 0.0027) were negatively correlated with consumers’ sensory liking. Most volatile compounds were not clearly related to the consumers’ sensory liking data except 5-methyl-2-furaldehyde (V7) and 3-methylphenol (V13) in the MFA map.

The current study provides comprehensive information concerning sensory profiles of cold brew. Information of cold brew coffee from this study can be used for coffee producers or marketing strategists.

## Figures and Tables

**Table 1:** Coffee samples information.

Sample Name	Category	Degree of Roasting (Coffee Variety)	Date of Roasting (year/month/day)	Purchased From
Blending	CB ^1^, CM ^2^	City (Brazil, Colombia, Ethiopia aricha, Guatemala)	16/07/28	Coffee loves him (Busan, Korea)
Colombia Narino Pasto Excelso	CB, CM	Medium	16/08/04	Koozberry (Busan, Korea)
Folgers classic roast	CB, CM	Medium		Lotte Mart (Seoul, Korea)
Kenya AA Kagumoini	CB, CM	Medium (Kenya)	16/08/04	Coffee loves him (Busan, Korea)
Barista rules cold brew (Maeil, Seoul, Korea)	RTD ^3^	Medium (El Salvador SHG)		GS Retail convenience store (Busan, Korea)
Cold brew by Babinski (Korea Yacult, Seoul, Korea)	RTD	Between medium and high (Brazil, Colombia, Ethiopia)		Korea Yacult delivery service (Busan, Korea)
French Café cold brew (Namyang, Seoul, Korea)	RTD	Unknown (Ethiopia (Yergacheffe, Sidamo blend))		GS Retail convenience store (Busan, Korea)
Starbucks (Seattle, WA, USA)	CS ^4^	Medium (Latin America & Africa blend)		Local Starbucks store (Busan, Korea)
Twosomeplace (Seoul, Korea)	CS	Unknown		Local Twosomeplace store (Busan, Korea)

^1^ CB stands for cold brewing; ^2^ CM stands for coffee maker brewing; ^3^ RTD stands for ready-to-drink; ^4^ CS stands for cold brew coffee purchased from coffee shop.

**Table 2:** Consumers’ coffee consumption behavior.

Category	Response (*n* = 120)	Response (%)
**Frequency of consuming hot (cold) black coffee**		
More than twice a day	6 (15)	5.0 (12.5)
Once a day	9 (37)	7.5 (30.8)
2 or 3 times a week	25 (39)	20.8 (32.5)
Once a week	18 (20)	15.0 (16.7)
Once a month	32 (5)	26.7 (4.2)
Once every three months	10 (2)	8.3 (1.7)
Once every six months	7 (2)	5.8 (1.7)
Do not drink	13 (0)	10.8 (0.0)
**Time of hot (cold) black coffee consumption**		
In the morning	36 (33)	30.0 (27.5)
In the afternoon	62 (77)	51.7 (64.2)
In the evening (after 6 pm)	21 (4)	17.5 (3.3)
Others	1 (6)	0.8 (5.0)
**Reason to drink cold black coffee (check-all-that-apply)**		
Cool	105	
Little bitter taste	15	
High amount of caffeine	14	
Good coffee flavor	44	
Others	0	
**Important aspects when select coffee (check-all-that-apply)**		
Taste	108	
Aroma	92	
Degree of bitterness	80	
Price	90	
Origin of coffee bean	11	
Brand	6	
Extraction method	10	
Fair trade	5	
Organic and environment-friendly	1	
Others	0	
**Type of favorite coffee**		
Coffee shop	104	86.7
Instant coffee	5	4.2
Bottled/Canned coffee	8	6.7
Vending machine coffee	0	0.0
Capsule coffee	3	2.5
Others	0	0.0
**Usually drink when**		
Talk with other people	15	12.5
After the meal	44	36.7
After the go to work/school	23	19.2
When distracted	32	26.7
Others	6	5.0

**Table 3:** Analysis of variance of consumers’ liking and perceived intensity for coffee samples.

Sample	Overall Liking	Bitterness Liking	Sourness Liking	CoffeeID Liking	Bitterness Intensity	Sourness Intensity	CoffeeID Intensity
BlendingCB	4.3 ^bcd^	4.5 ^d^	4.5 ^d^	4.9 ^cde^	4.9 ^d^	5.7 ^b^	5.6 ^bcd^
ColombiaCB	3.6 ^efg^	3.5 ^f^	3.5 ^f^	4.4 ^def^	4.6 ^de^	6.6 ^a^	5.2 ^ef^
FolgersCB	4.1 ^cde^	4.5 ^cd^	4.5 ^cd^	4.8 ^cde^	5.8 ^bc^	3.1 ^c^	5.5 ^cde^
KenyaCB	3.5 ^fgh^	3.4 ^f^	3.4 ^f^	4.4 ^ef^	4.4 ^ef^	6.8 ^a^	5.4 ^def^
BlendingCM	4.6 ^bcd^	4.5 ^de^	4.5 ^de^	5.0 ^cde^	4.9 ^d^	5.8 ^b^	5.2 ^ef^
ColombiaCM	3.5 ^gh^	3.5 ^f^	3.5 ^f^	4.9 ^c^	4.9 ^d^	6.9 ^a^	5.4 ^def^
FolgersCM	3.2 ^gh^	4.0 ^e^	4.0 ^e^	4.4 ^ef^	6.8 ^a^	5.4 ^bc^	6.0 ^b^
KenyaCM	3.1 ^h^	3.1 ^f^	3.1 ^f^	4.1 ^f^	5.0 ^d^	6.9 ^a^	5.8 ^bc^
BabinskiRTD	5.5 ^a^	5.3 ^ab^	5.3 ^ab^	5.8 ^a^	3.9 ^f^	5.2 ^c^	5.2 ^ef^
BaristaRTD(A)	5.5 ^a^	5.8 ^a^	5.6 ^a^	5.7 ^ab^	4.2 ^ef^	3.3 ^f^	5.2 ^ef^
BaristaRTD(B)	5.8 ^a^	5.8 ^a^	5.7 ^a^	5.7 ^ab^	4.1 ^ef^	3.4 ^f^	5.0 ^f^
FrenchCaféRTD	4.7 ^bc^	5.0 ^bc^	5.0 ^bc^	5.2 ^bc^	4.1 ^f^	4.7 ^d^	5.5 ^cde^
StarbucksCS	4.1 ^def^	4.6 ^cd^	4.6 ^cd^	5.0 ^c^	6.3 ^b^	5.0 ^cd^	6.0 ^ab^
TwosomeplaceCS	4.8 ^b^	5.2 ^b^	5.2 ^b^	5.1 ^c^	5.7 ^c^	4.1 ^e^	6.4 ^a^
*p*-value	<0.0001	<0.0001	<0.0001	<0.0001	<0.0001	<0.0001	<0.0001
Least significant difference (LSD)	0.52	0.47	0.44	0.49	0.47	0.46	0.41
